# Complete genome sequences of two novel *Ralstonia* jumbo phages isolated from leaf litter compost

**DOI:** 10.1007/s00705-024-06162-9

**Published:** 2024-11-01

**Authors:** Ryota Sasaki, Shuhei Miyashita, Hideki Takahashi

**Affiliations:** Graduate School of Agricultural Science, Tohoku University, Sendai, Miyagi 980-0845 Japan

## Abstract

Two *Ralstonia* phages, FLC1-1B and FLC4-3B, were isolated from leaf litter compost, using *Ralstonia pseudosolanacearum*, which is a causal agent of bacterial wilt disease, as a host. The genomic DNA sequences of FLC1-1B and FLC4-3B were determined and found to be 290,008 bp and 291,257 bp in length, respectively, and they were therefore classified as jumbo phages. However, they did not show high similarity to any jumbo phage genomic sequence in the NCBI nt database. The closest hit in a BLAST search was the jumbo phage ripduovirus RP12, with only 35% coverage and 77% sequence identity, whereas the FLC1-1B and FLC4-3B sequences were 99.0% identical. Based on these findings, FLC1-1B and FLC4-3B should be classified as members of a new genus in the order *Caudoviricetes*. FLC4-3B was found to suppress wilt disease in tomato plants, suggesting that it has potential as a biocontrol agent for managing *R*. *pseudosolanacearum* infections.

**Supplementary Information:**

The online version contains supplementary material available at 10.1007/s00705-024-06162-9.

*Ralstonia solanacearum*, *R*. *pseudosolanacearum*, and *R*. *syzygii* are well-known causal agents of bacterial wilt, which results in severe agricultural losses and are therefore considered important phytopathogenic bacteria [1, 2]. Because bacteriophages (phages) can infect and lyse *Ralstonia* spp., lytic phages have attracted considerable attention as potential biocontrol agents for managing bacterial wilt disease [3]. Various phages capable of infecting *Ralstonia* spp. have been isolated [3]. Six of them – RSL1, RSF1, RSL2, RP12, RP31, and RsoM2USA – are jumbo phages with genomes larger than 200 kbp, and they have been reported to have a broad host range [4–8]. Furthermore, it has been reported that the *Burkholderia* jumbo phages FLC6, FLC8, and FLC9, which were isolated from leaf litter compost, have the ability to lyse multiple isolates of *R*. *pseudosolanacearum* [9–11]. To identify jumbo phages that can be used as biocontrol agents with lytic potential against a broad range of pathogenic *Ralstonia* spp. in order to control bacterial wilt disease, further isolation of *Ralstonia* jumbo phages is necessary. In this study, two novel *Ralstonia* jumbo phages – FLC1-1B and FLC4-3B – were isolated from leaf litter compost, and their complete genomes were sequenced for proteomic tree analysis. Their host range, virion structure, and ability to suppress bacterial wilt disease were also examined.

The methods used for phage isolation, phage DNA extraction, electron microscopy, and host range analysis are described in the online Supplementary Material. Electron microscopy of the phage isolates revealed that they both have an icosahedral head and a contractile tail (Supplementary Fig. S1), suggesting that they belong to the order *Caudoviricetes*.

The procedures for phage DNA library construction, DNA sequencing, and *in silico* data analysis are also described in the online Supplementary Material. Analysis of the NGS read data used for assembly of the genome sequences indicated that the sequence coverage for FLC1-1B and FLC4-3B was 140 and 110, respectively. The complete genome sequence of FLC1-1B is 290,008 bp in length (G+C content, 56.00%), and that of FLC4-3B is 291,257 bp in length (G+C content = 55.95%), and they can therefore both be classified as jumbo phages. A BLASTn search of the NCBI nt database using the complete genome sequences of FLC1-1B and FLC4-3B indicated that the closest relative was *Ralstonia* phage RP12 (accession number NC_041911.1), which belongs to the genus *Ripduovirus* of the order *Caudoviricetes*. RP12 is a KZ-related phage that has a circularly permuted and terminally redundant structure [8]. Since the nucleotide sequence reads in this study were assembled into a circular contig and the reads were evenly mapped across the entire genome (data not shown), both FLC1-1B and FLC4-3B appear to have a circularly permuted and terminally redundant structure, like RP12. For the purpose of comparison, the nucleotide numbering and orientation of the FLC1-1B and FLC4-3B genome sequences were defined based on an alignment with the RP12 genome sequence from the GenBank/EMBL/DDBJ database (Fig. 1A). The query coverage and identity between RP12 and FLC1-1B were 35% and 77.40%, respectively. The query coverage and identity between FLC4-3B with RP12 were 35% and 77.38%, respectively. The genome sequences of FLC1-1B and FLC4-3B were found to be 99.0% identical to each other. According to a recent proposal for genome-based phage taxonomy [12], if the product of the query coverage value and the identity value obtained for a full-genome sequence comparison is less than 0.70, the viruses should be classified as members of different genera. Here, the product of the query coverage and identity values of RP12 and FLC1-1B was 0.2709 (0.35 × 0.774 = 0.2709), and that of RP12 and FLC4-3B was 0.2708 (0.35 × 0.774 = 0.2708). Because both of these values are below 0.70, FLC1-1B and FLC4-3B should be classified as members of a new genus. The numbers of predicted open reading frames (ORFs) in the FLC1-1B and FLC4-3B genomes were 309 and 310, respectively (Fig. 1A and Supplementary Tables S1 and S2). A total of 54 ORFs in each genome were annotated based on the annotation of RP12 (Fig. 1A and Supplementary Tables S1 and S2). Five tRNA genes were detected in both genomes, whereas no rRNA genes were identified (Fig. 1B). A proteomic tree analysis based on the amino acid sequences of all of the proteins encoded by the phage genome indicated that FLC1-1B and FLC4-3B belong to the same clade (Fig. 2). This was also supported by phylogenetic analysis based on amino acid sequences of the terminase large subunit and SbcC (Supplementary Figs. S1 and S2). These results further support the classification of FLC1-1B and FLC4-3B as members of a new genus in the order *Caudoviricetes*.

**Fig. 1:**
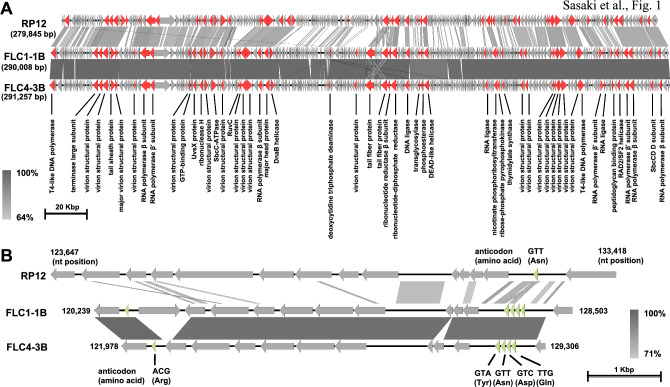
Comparison of the RP12, FLC1-1B, and FLC4-3B genomes. (A) Comparison of the complete genomes of RP12 (279,845 bp), FLC1-1B (290,008 bp), and FLC4-3B (291,257 bp). Red arrows represent annotated ORFs, and the putative functions of their products are shown. (B). Partial genome comparison showing the region in which tRNAs are encoded (nt 123,647 to 133,418 of RP12, nt 120,239 to 128,503 of FLC1-1B, and nt 121,978 to 129,306 FLC4-3B). Light green triangles indicate tRNA genes, and the anticodon and corresponding amino acid are shown. In A and B, gray arrows represent ORFs encoding hypothetical proteins. Shades of gray indicate the DNA sequence identity determined using BLASTn

**Fig. 2:**
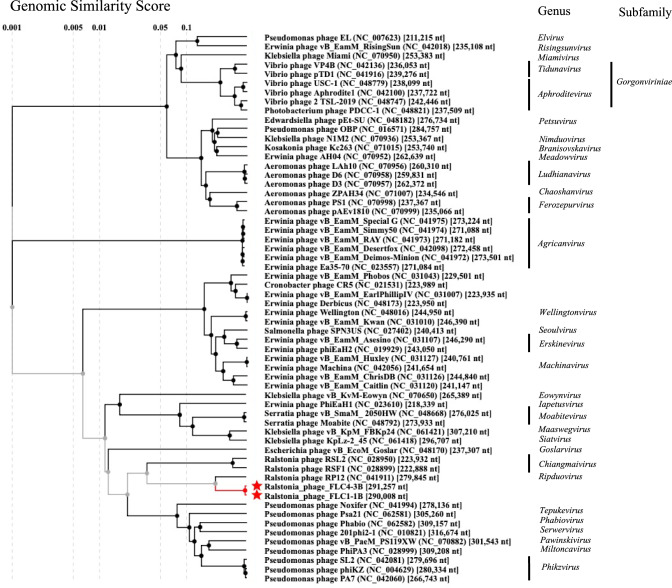
Proteomic tree of *Ralstonia* phages FLC1-1B and FLC4-3B and other jumbo phages. The amino acid sequences of all of the proteins encoded by the phage genomes were used for the analysis. FLC1-1B and FLC4-3B are indicated by red stars. The ICTV master species list 2022 MSL38 ver. 3 was referenced for taxonomy

A host range analysis using nine isolates of *R*. *pseudosolanacearum* and three isolates of *R*. *syzygii* subsp. *indonesiensis* (Supplementary Table S3) revealed that FLC1-1B and FLC4-3B were able to lyse five and three, respectively, of the nine *R*. *pseudosolanacearum* isolates, while both phages were unable to lyse any of the *R*. *syzygii* subsp. *indonesiensis* isolates (Supplementary Table S4). Since multiple pathotypes of *Ralstonia* spp seem to inhabit the soil of agricultural sites, jumbo phages with a broad host range might have advantages as biocontrol agents against bacterial wilt disease. Treatment of tomato nursery plants with FLC4-3B followed by inoculation with *R*. *pseudosolanacearum* significantly suppressed the occurrence of wilt disease (Supplementary Fig. S3), suggesting its potential as a biocontrol agent. However, the functions of the many proteins encoded by FLC1-1B and FLC4-3B need to be identified in order to determine whether these phages can undergo a lysogenic cycle and integrate into the host genome. Clarifying this issue will help to determine whether these jumbo phages are appropriate for use as biocontrol agents for managing bacterial wilt disease in cultivated tomato plants.

**Nucleotide sequence accession numbers** The GenBank/EMBL/DDBJ accession numbers for *Ralstonia* phages FLC1-1B and FLC4-3B are LC819005 and LC819007, respectively.

## Supplementary Information

Below is the link to the electronic supplementary material.

Supplementary file1 (DOCX 11395 KB)

## Data Availability

The data supporting the findings of this study are available in the GenBank database.
