# Supplementary material for: Complete genome sequences of two novel Ralstonia jumbo phages isolated from leaf litter compost
Source: Arch Virol. 2024 Nov 1;169(11):235. doi: 10.1007/s00705-024-06162-9 (PMC11530567; doi:10.1007/s00705-024-06162-9)
Supplement: Supplementary file 1 — Supplementary file1 (DOCX 11395 KB) [file 705_2024_6162_MOESM1_ESM.docx]

**Online Supplementary Material**

material.

**Phage isolation, DNA extraction, electron microscopy observation, and host range analysis**

Phages were isolated from leaf litter compost, using the method described by Sasaki et al. (2021) [1] with some modifications. The method involves casamino acid-peptone-glucose (CPG) medium [1.0% (w/v) peptone, 0.1% (w/v) casamino acid, and 0.5% (w/v) glucose] and *R. pseudosolanacearum* strain MAFF106603 as the host, with an incubation temperature of 28°C. Phage multiplication and purification were performed using *R. pseudosolanacearum* MAFF106611. The extraction of phage genome DNA, the determination of complete phage genomes using MiSeq (Illumina, San Diego, CA), and the observation of phage particles with transmission electron microscopy were performed as previously described [1]. The host ranges of the isolated phages were analyzed using the spot test with nine *R*. *pseudosolanacearum* strains and three *R*. *syzygii* subsp. *indonesiensis* strains, which were supplied from public resource stock center GeneBank (NARO, Tsukuba, Japan) (Table S3).

***In silico* analyses**

Filtered reads were assembled using SPAdes ver. 3.13.0 [2]. The obtained sequences were used for a homologous search with blastn of the NCBI nt database. GeneMarkS ver. 4.28 [3] was used for the prediction of open reading frames (ORFs). An amino acid sequence of the product from each predicted ORF was used as a query for the blastp search (e-value ≤ 1e−5) with NCBI nr limited in *Caudoviricetes* and annotated following the description of the homologs in the Ralstonia phage RP12(NC_041911.1). tRNA and rRNA sequences were detected using tRNAscan-SE ver. 2.0 [4], ARAGON ver1.2.41 [5] and rnammer ver. 1.2 [6], respectively. The complete genome comparison was visualized using EasyFig ver. 2.2.2 [7]. Amino acid sequences of proteins encoded on all predicted phage open reading frames (ORFs) were used for proteomic tree analysis, which was performed using ViPTree ver. 4.0 [8]. A phylogenetic tree of the terminase large subunit gene and the SbcC gene was constructed and visualized using the “one click” mode of phylogeny.fr (<https://www.phylogeny.fr>) [9, 10], based on the amino acid sequences as follows. Multiple alignment was performed using MUSCLE [11], alignment curation was performed using Gblocks [12], the sequence-based maximum likelihood tree was constructed using the PhyML [13, 14], and the tree was drawn using TreeDyn [15].

**Ralstonia phage treatment, inoculation with *R. pseudosolanacearium*, and wilt disease assessment**

Bacteria were cultured at 28°C in liquid CPG medium containing 0.001% tetrazolium chloride for 48 h on a rotary shaker [16]. Subsequently, the bacterial cells were collected by centrifugation and resuspended in distilled sterilized water to a final density of 1×10^7^ cfu/mL. The roots of five-leaf-stage tomato nursery plants were soaked in Ralstonia phage FLC4-3B solution (1×10^7^ pfu/mL) or in diluted CPG medium of the same strength as that used to prepare the FLC4-3B solution, for 1 h at 25°C. After soaking the roots of tomato plants with sterilized distilled water five times, the phage-treated plants were inoculated with *Ralstonia pseudosolanacearium* MAFF106611 (1×10^7^ cfu/mL) by soaking their roots in the bacterial solution. The inoculated plants were cultivated in a growth chamber at 30°C under 16-h light conditions (70 μmol/m^2^/s). At 10 days after inoculation with *R. pseudosolanacearium*, the disease severity was categorized on a 0–4 non-parametric scale (0, no wilting; 1, 1%–25% wilting; 2, 26%–50% wilting; 3, 51%–75% wilting; 4, 76%–100% wilting or dead) and the wilt disease severity index (DSI) was calculated [17]. Each assay was repeated in three successive trials, and six plants were inoculated in each experiment. Wilcoxon’s two-sample test was used to check for significant differences in disease severity (α = 0.05).

**References**

1. Sasaki R, Miyashita S, Ando S, Ito K, Fukuhara T, Takahashi H (2021) Isolation and characterization of a novel jumbo phage from leaf litter compost and its suppressive effect on rice seedling. Viruses 13:591

2. Nurk S, Bankevich A, Antipov D, Gurevich A, Korobeynikov A, Lapidus A, Prjibelsky A, Pyshkin A, Sirotkin A, Sirotkin Y, Stepanauskas R, McLean J, Lasken R, Clingenpeel S, Woyke T, Tesler G, Alekseyev M, Pevzner P (2013) Assembling genomes and mini-metagenomes from highly chimeric reads. In: Deng M, Jiang R, Sun F, Zhang X (eds) Research in Computational Molecular Biology. RECOMB 2013. Lecture Notes in Computer Science. Springer, Berlin, Heidelberg, pp 158-170

3. Besemer J, Lomsadze A, Borodovsky M (2001) GeneMarkS: a self-training method for prediction of gene starts in microbial genomes. Implications for finding sequence motifs in regulatory regions. Nucleic Acids Res 29:2607-2618

4. Chan PP, Lin BY, Mak AJ, Lowe TM (2021) TRNAscan-SE 2.0: Improved detection and functional classification of transfer RNA genes. Nucleic Acids Res 49:9077-9096

5. Lagesen K, Hallin P, Rødland EA, Stærfeldt HH, Rognes T, Ussery DW (2007) RNAmmer: Consistent and rapid annotation of ribosomal RNA genes. Nucleic Acids Res 35:3100-3108

6. Laslett D, Canback B (2004) ARAGORN, a program for the detection of transfer RNA and transfer-messenger RNA genes in nucleotide sequences. Nucleic Acids Res 32:11-16

7. Sullivan MJ, Petty NK, Beatson SA (2011) Easyfig: A genome comparison visualizer. Bioinformatics 27:1009-1010

8. Nishimura Y, Yoshida T, Kuronishi M, Uehara H, Ogata H, Goto S (2017) ViPTree: The viral proteomic tree server. Bioinformatics 33:2379-2380

9. Dereeper A, Guignon V, Blanc G, Audic S, Buffet S, Chevenet F, Dufayard JF, Guindon S, Lefort V, Lescot M, Claverie JM, Gascuel O (2008) Phylogeny.fr: robust phylogenetic analysis for the non-specialist. Nucleic Acids Res 36:465-469

10. Dereeper A, Audic S, Claverie JM, Blanc G (2010) BLAST-EXPLORER helps you building datasets for phylogenetic analysis. BMC Evol Biol 10:8-13

11. Edgar RC (2004) MUSCLE: Multiple sequence alignment with high accuracy and high throughput. Nucleic Acids Res 32:1792-1797

12. Castresana J (2000) Selection of conserved blocks from multiple alignments for their use in phylogenetic analysis. Mol Biol Evol 17:540-552

13. Guindon S, Gascuel O (2003) A simple, fast, and accurate algorithm to estimate large phylogenies by maximum likelihood. Syst Biol 52:696-704

14. Anisimova M, Gascuel O (2006) Approximate likelihood-ratio test for branches: A fast, accurate, and powerful alternative. Syst Biol 55:539-552

15. Chevenet F, Brun C, Bañuls AL, Jacq B, Christen R (2006) TreeDyn: Towards dynamic graphics and annotations for analyses of trees. BMC Bioinformatics 7:1-9

16. Hendrick C, Sequeira L (1984) Lipopolysaccharide-defective mutants of the wilt pathogen *Pseudomonas solanacearum*. Appl Environ Microbiol 48:94-101

17. Roberts DP, Denny TP, Schell MA (1988) Cloning of the egl gene of *Pseudomonas solanacearum* and analysis of its role in phytopathogenicity. J Bacteriol 170:1445-1451

**Fig. S1** Phage particles observed with transmission electron microscopy. Intact particles of (**a**) FLC1-1B and (**b**) FLC4-3B. Particles of (**c**) FLC1-1B and (**d**) FLC4-3B with a contracted tail. The bars represent a length of 100 nm.

**Fig. S2** Phylogenetic tree based on the amino acid sequences of (**a**) the terminase large subunit and (**b**) SbcC. Escherichia phage T4 was used as an outgroup. The Ralstonia phages FLC1-1B and FLC4-3B are shown in blue. Red numbers show the branch support value calculated using an approximate likelihood ratio test [1].

[1] Anisimova M, Gascuel O (2006) Approximate likelihood-ratio test for branches: A fast, accurate, and powerful alternative. Syst Biol 55:539-552

**Fig. S3** Assessment of the suppression of wilt disease by treatment with the Ralstonia phage FLC4-3B. (**a**) Photograph showing representative wilt disease symptoms at 10 days after inoculation with *R. pseudosolanacearum*. The roots of five-leaf-stage tomato nursery plants were soaked in Ralstonia phage solution (1×10^7^ pfu/mL) (FLC4-3B) or in diluted CPG medium of the same strength as that used to prepare the FLC4-3B solution (Control), for 1 h at 25°C, followed by inoculation with *Ralstonia pseudosolanacearum* MAFF106611 (1×10^7^ cfu/mL). (**b**) The proportion of wilt disease severity index (DSI) scores for *R. pseudosolanacearum*-infected plants that were immersed in Ralstonia phage solution or in diluted CPG medium as a control was assessed. The DSI scores are shown above the bar chart. The disease severity was categorized on a 0–4 non-parametric scale (0, no wilting; 1, 1%–25% wilting; 2, 26%–50% wilting; 3, 51%–75% wilting; 4, 76%–100% wilting or dead) and the DSI was calculated. Wilcoxon’s two-sample test was used to check for significant differences in disease severity (α = 0.05).

| **Table S1.** Gene products encoded on genomic DNA of FLC1-1B and their closest hit searched with blastp in NCBI nr limited in *Caudoviricetes*. | | |
| --- | --- | --- |
| Gene products | The closest hits^1^ |  |
|  | Protein names [Virus strain] | Accession numbers |
| 1 | putative T4-like DNA polymerase [Ralstonia phage RP12] | YP_009598696.1 |
| 2 | no hit |  |
| 3 | hypothetical protein FDH28_gp004 [Ralstonia phage RP12] | YP_009598697.1 |
| 4 | hypothetical protein FDH28_gp005 [Ralstonia phage RP12] | YP_009598698.1 |
| 5 | hypothetical protein FDH28_gp006 [Ralstonia phage RP12] | YP_009598699.1 |
| 6 | hypothetical protein FDH28_gp007 [Ralstonia phage RP12] | YP_009598700.1 |
| 7 | hypothetical protein FDH28_gp008 [Ralstonia phage RP12] | YP_009598701.1 |
| 8 | hypothetical protein FDH28_gp009 [Ralstonia phage RP12] | YP_009598702.1 |
| 9 | hypothetical protein FDH28_gp010 [Ralstonia phage RP12] | YP_009598703.1 |
| 10 | no hit |  |
| 11 | hypothetical protein FDH28_gp011 [Ralstonia phage RP12] | YP_009598704.1 |
| 12 | hypothetical protein FDH28_gp012 [Ralstonia phage RP12] | YP_009598705.1 |
| 13 | hypothetical protein FDH28_gp013 [Ralstonia phage RP12] | YP_009598706.1 |
| 14 | hypothetical protein FDH28_gp014 [Ralstonia phage RP12] | YP_009598707.1 |
| 15 | no hit |  |
| 16 | hypothetical protein FDH28_gp016 [Ralstonia phage RP12] | YP_009598709.1 |
| 17 | no hit |  |
| 18 | hypothetical protein FDH28_gp017 [Ralstonia phage RP12] | YP_009598710.1 |
| 19 | hypothetical protein FDH28_gp018 [Ralstonia phage RP12] | YP_009598711.1 |
| 20 | hypothetical protein FDH28_gp019 [Ralstonia phage RP12] | YP_009598712.1 |
| 21 | hypothetical protein FDH28_gp020 [Ralstonia phage RP12] | YP_009598713.1 |
| 22 | hypothetical protein FDH28_gp021 [Ralstonia phage RP12] | YP_009598714.1 |
| 23 | hypothetical protein FDH28_gp022 [Ralstonia phage RP12] | YP_009598715.1 |
| 24 | hypothetical protein FDH28_gp023 [Ralstonia phage RP12] | YP_009598716.1 |
| 25 | putative terminase, large subunit [Ralstonia phage RP12] | YP_009598717.1 |
| 26 | putative virion structural protein [Ralstonia phage RP12] | YP_009598718.1 |
| 27 | putative virion structural protein [Ralstonia phage RP12] | YP_009598719.1 |
| 28 | hypothetical protein FDH28_gp027 [Ralstonia phage RP12] | YP_009598720.1 |
| 29 | putative tail shealth protein [Ralstonia phage RP12] | YP_009598722.1 |
| 30 | putative major virion structural protein [Ralstonia phage RP12] | YP_009598723.1 |
| 31 | no hit |  |
| 32 | no hit |  |
| 33 | hypothetical protein FDH28_gp031 [Ralstonia phage RP12] | YP_009598724.1 |
| 34 | no hit |  |
| 35 | hypothetical protein FDH28_gp032 [Ralstonia phage RP12] | YP_009598725.1 |
| 36 | hypothetical protein FDH28_gp034 [Ralstonia phage RP12] | YP_009598727.1 |
| 37 | hypothetical protein FDH28_gp036 [Ralstonia phage RP12] | YP_009598729.1 |
| 38 | putative virion structural protein [Ralstonia phage RP12] | YP_009598730.1 |
| 39 | hypothetical protein FDH28_gp038 [Ralstonia phage RP12] | YP_009598731.1 |
| 40 | hypothetical protein FDH28_gp039 [Ralstonia phage RP12] | YP_009598732.1 |
| 41 | hypothetical protein FDH28_gp040 [Ralstonia phage RP12] | YP_009598733.1 |
| 42 | putative RNA polymerase beta subunit [Ralstonia phage RP12] | YP_009598734.1 |
| 43 | putative RNA polymerase beta prime subunit [Ralstonia phage RP12] | YP_009598735.1 |
| 44 | hypothetical protein FDH28_gp043 [Ralstonia phage RP12] | YP_009598736.1 |
| 45 | hypothetical protein FDH28_gp044 [Ralstonia phage RP12] | YP_009598737.1 |
| 46 | hypothetical protein FDH28_gp046 [Ralstonia phage RP12] | YP_009598739.1 |
| 47 | hypothetical protein FDH28_gp047 [Ralstonia phage RP12] | YP_009598740.1 |
| 48 | hypothetical protein FDH28_gp048 [Ralstonia phage RP12] | YP_009598741.1 |
| 49 | hypothetical protein FDH28_gp049 [Ralstonia phage RP12] | YP_009598742.1 |
| 50 | no hit |  |
| 51 | hypothetical protein FDH28_gp051 [Ralstonia phage RP12] | YP_009598744.1 |
| 52 | hypothetical protein FDH28_gp052 [Ralstonia phage RP12] | YP_009598745.1 |
| 53 | hypothetical protein FDH28_gp053 [Ralstonia phage RP12] | YP_009598746.1 |
| 54 | putative virion structural protein [Ralstonia phage RP12] | YP_009598748.1 |
| 55 | putative GTP-binding protein [Ralstonia phage RP12] | YP_009598749.1 |
| 56 | no hit |  |
| 57 | hypothetical protein FDH28_gp058 [Ralstonia phage RP12] | YP_009598751.1 |
| 58 | putative UvsX protein [Ralstonia phage RP12] | YP_009598752.1 |
| 59 | hypothetical protein FDH28_gp060 [Ralstonia phage RP12] | YP_009598753.1 |
| 60 | hypothetical protein FDH28_gp061 [Ralstonia phage RP12] | YP_009598754.1 |
| 61 | putative ribonuclease H [Ralstonia phage RP12] | YP_009598755.1 |
| 62 | hypothetical protein FDH28_gp063 [Ralstonia phage RP12] | YP_009598756.1 |
| 63 | hypothetical protein FDH28_gp064 [Ralstonia phage RP12] | YP_009598757.1 |
| 64 | putative virion structural protein [Ralstonia phage RP12] | YP_009598758.1 |
| 65 | hypothetical protein FDH28_gp066 [Ralstonia phage RP12] | YP_009598759.1 |
| 66 | hypothetical protein FDH28_gp067 [Ralstonia phage RP12] | YP_009598760.1 |
| 67 | putative SbcC-ATPase [Ralstonia phage RP12] | YP_009598762.1 |
| 68 | no hit |  |
| 69 | no hit |  |
| 70 | no hit |  |
| 71 | hypothetical protein FDH28_gp070 [Ralstonia phage RP12] | YP_009598763.1 |
| 72 | putative virion structural protein [Ralstonia phage RP12] | YP_009598764.1 |
| 73 | hypothetical protein FDH28_gp072 [Ralstonia phage RP12] | YP_009598765.1 |
| 74 | hypothetical protein FDH28_gp073 [Ralstonia phage RP12] | YP_009598766.1 |
| 75 | no hit |  |
| 76 | hypothetical protein FDH28_gp082 [Ralstonia phage RP12] | YP_009598775.1 |
| 77 | no hit |  |
| 78 | hypothetical protein FDH28_gp084 [Ralstonia phage RP12] | YP_009598777.1 |
| 79 | hypothetical protein FDH28_gp085 [Ralstonia phage RP12] | YP_009598778.1 |
| 80 | putative crossover junction endodeoxyribonuclease RuvC [Ralstonia phage RP12] | YP_009598779.1 |
| 81 | putative virion structural protein [Ralstonia phage RP12] | YP_009598780.1 |
| 82 | putative virion structural protein [Ralstonia phage RP12] | YP_009598781.1 |
| 83 | putative virion structural protein [Ralstonia phage RP12] | YP_009598782.1 |
| 84 | no hit |  |
| 85 | hypothetical protein FDH28_gp091 [Ralstonia phage RP12] | YP_009598784.1 |
| 86 | putative RNA polymerase beta subunit [Ralstonia phage RP12] | YP_009598785.1 |
| 87 | hypothetical protein FDH28_gp093 [Ralstonia phage RP12] | YP_009598786.1 |
| 88 | putative major head protein [Ralstonia phage RP12] | YP_009598787.1 |
| 89 | hypothetical protein FDH28_gp095 [Ralstonia phage RP12] | YP_009598788.1 |
| 90 | putative DnaB helicase [Ralstonia phage RP12] | YP_009598789.1 |
| 91 | no hit |  |
| 92 | hypothetical protein FDH28_gp103 [Ralstonia phage RP12] | YP_009598796.1 |
| 93 | hypothetical protein FDG98_gp017 [Pseudomonas phage pf16] | YP_009595471.1 |
| 94 | no hit |  |
| 95 | hypothetical protein FDH28_gp100 [Ralstonia phage RP12] | YP_009598793.1 |
| 96 | no hit |  |
| 97 | no hit |  |
| 98 | hypothetical protein FDH28_gp105 [Ralstonia phage RP12] | YP_009598798.1 |
| 99 | hypothetical protein CF95_gp196 [Erwinia phage PhiEaH1] | YP_009010249.1 |
| 100 | hypothetical protein FDH28_gp106 [Ralstonia phage RP12] | YP_009598799.1 |
| 101 | no hit |  |
| 102 | no hit |  |
| 103 | no hit |  |
| 104 | no hit |  |
| 105 | no hit |  |
| 106 | no hit |  |
| 107 | no hit |  |
| 108 | hypothetical protein FDH28_gp111 [Ralstonia phage RP12] | YP_009598804.1 |
| 109 | no hit |  |
| 110 | no hit |  |
| 111 | hypothetical protein FDH28_gp114 [Ralstonia phage RP12] | YP_009598807.1 |
| 112 | no hit |  |
| 113 | hypothetical protein FDH28_gp115 [Ralstonia phage RP12] | YP_009598808.1 |
| 114 | hypothetical protein FDH28_gp116 [Ralstonia phage RP12] | YP_009598809.1 |
| 115 | hypothetical protein FDH28_gp117 [Ralstonia phage RP12] | YP_009598810.1 |
| 116 | hypothetical protein FDH28_gp118 [Ralstonia phage RP12] | YP_009598811.1 |
| 117 | hypothetical protein FDH28_gp119 [Ralstonia phage RP12] | YP_009598812.1 |
| 118 | no hit |  |
| 119 | virion structural protein [Pseudomonas phage Psa21] | YP_010347966.1 |
| 120 | hypothetical protein FDH28_gp121 [Ralstonia phage RP12] | YP_009598814.1 |
| 121 | hypothetical protein FDH28_gp120 [Ralstonia phage RP12] | YP_009598813.1 |
| 122 | no hit |  |
| 123 | hypothetical protein FDH28_gp124 [Ralstonia phage RP12] | YP_009598817.1 |
| 124 | hypothetical protein FDH28_gp125 [Ralstonia phage RP12] | YP_009598818.1 |
| 125 | no hit |  |
| 126 | no hit |  |
| 127 | no hit |  |
| 128 | hypothetical protein FDH28_gp130 [Ralstonia phage RP12] | YP_009598823.1 |
| 129 | no hit |  |
| 130 | hypothetical protein FDH28_gp289 [Ralstonia phage RP12] | YP_009598825.1 |
| 131 | hypothetical protein FDH28_gp097 [Ralstonia phage RP12] | YP_009598790.1 |
| 132 | hypothetical protein FDH28_gp288 [Ralstonia phage RP12] | YP_009598826.1 |
| 133 | hypothetical protein CF95_gp037 [Erwinia phage PhiEaH1] | YP_009010090.1 |
| 134 | putative deoxycytidine triphosphate deaminase [Ralstonia phage RP12] | YP_009598827.1 |
| 135 | no hit |  |
| 136 | no hit |  |
| 137 | hypothetical protein FDH28_gp285 [Ralstonia phage RP12] | YP_009598829.1 |
| 138 | hypothetical protein FDH28_gp284 [Ralstonia phage RP12] | YP_009598830.1 |
| 139 | hypothetical protein FDH28_gp277 [Ralstonia phage RP12] | YP_009598837.1 |
| 140 | no hit |  |
| 141 | no hit |  |
| 142 | no hit |  |
| 143 | no hit |  |
| 144 | no hit |  |
| 145 | no hit |  |
| 146 | no hit |  |
| 147 | hypothetical protein MZD04_gp268 [Pseudomonas phage Psa21] | YP_010347817.1 |
| 148 | no hit |  |
| 149 | putative concanavalin A-like protein [Ralstonia phage RSF1] | YP_009207894.1 |
| 150 | no hit |  |
| 151 | hypothetical protein AVU38_gp183 [Ralstonia phage RSL2] | YP_009213032.1 |
| 152 | hypothetical protein AVU38_gp184 [Ralstonia phage RSL2] | YP_009213033.1 |
| 153 | hypothetical protein AVU38_gp185 [Ralstonia phage RSL2] | YP_009213034.1 |
| 154 | no hit |  |
| 155 | hypothetical protein FDI21_gp137 [Pseudomonas phage Noxifer] | YP_009609052.1 |
| 156 | putative tail fiber protein [Ralstonia phage RP12] | YP_009598846.1 |
| 157 | hypothetical protein FDH28_gp267 [Ralstonia phage RP12] | YP_009598847.1 |
| 158 | hypothetical protein FDH28_gp271 [Ralstonia phage RP12] | YP_009598843.1 |
| 159 | no hit |  |
| 160 | hypothetical protein FDH28_gp269 [Ralstonia phage RP12] | YP_009598845.1 |
| 161 | putative tail fiber protein [Ralstonia phage RP12] | YP_009598846.1 |
| 162 | putative ribonucleotide reductase of class Ia (Aerobic), beta subunit [Ralstonia phage RP12] | YP_009598848.1 |
| 163 | putative ribonucleoside-diphosphate reductase [Ralstonia phage RP12] | YP_009598849.1 |
| 164 | no hit |  |
| 165 | hypothetical protein FDH28_gp263 [Ralstonia phage RP12] | YP_009598851.1 |
| 166 | hypothetical protein FDH28_gp262 [Ralstonia phage RP12] | YP_009598852.1 |
| 167 | hypothetical protein FDH28_gp261 [Ralstonia phage RP12] | YP_009598853.1 |
| 168 | no hit |  |
| 169 | no hit |  |
| 170 | putative DNA ligase [Ralstonia phage RP12] | YP_009598854.1 |
| 171 | hypothetical protein FDH28_gp259 [Ralstonia phage RP12] | YP_009598855.1 |
| 172 | no hit |  |
| 173 | no hit |  |
| 174 | hypothetical protein FDH28_gp256 [Ralstonia phage RP12] | YP_009598858.1 |
| 175 | putative transglycosylase [Ralstonia phage RP12] | YP_009598859.1 |
| 176 | putative phosphoesterase [Ralstonia phage RP12] | YP_009598861.1 |
| 177 | no hit |  |
| 178 | no hit |  |
| 179 | putative DEAD-like helicase [Ralstonia phage RP12] | YP_009598862.1 |
| 180 | no hit |  |
| 181 | no hit |  |
| 182 | no hit |  |
| 183 | no hit |  |
| 184 | hypothetical protein FDH28_gp245 [Ralstonia phage RP12] | YP_009598869.1 |
| 185 | no hit |  |
| 186 | hypothetical protein FDH28_gp243 [Ralstonia phage RP12] | YP_009598871.1 |
| 187 | hypothetical protein FDH28_gp242 [Ralstonia phage RP12] | YP_009598872.1 |
| 188 | no hit |  |
| 189 | no hit |  |
| 190 | hypothetical protein FDH28_gp241 [Ralstonia phage RP12] | YP_009598873.1 |
| 191 | hypothetical protein FDH28_gp240 [Ralstonia phage RP12] | YP_009598874.1 |
| 192 | no hit |  |
| 193 | no hit |  |
| 194 | no hit |  |
| 195 | hypothetical protein FDH28_gp237 [Ralstonia phage RP12] | YP_009598877.1 |
| 196 | hypothetical protein FDH28_gp236 [Ralstonia phage RP12] | YP_009598878.1 |
| 197 | no hit |  |
| 198 | hypothetical protein FDH28_gp234 [Ralstonia phage RP12] | YP_009598880.1 |
| 199 | hypothetical protein FDH28_gp233 [Ralstonia phage RP12] | YP_009598881.1 |
| 200 | hypothetical protein FDH28_gp232 [Ralstonia phage RP12] | YP_009598882.1 |
| 201 | no hit |  |
| 202 | hypothetical protein FDH28_gp231 [Ralstonia phage RP12] | YP_009598883.1 |
| 203 | no hit |  |
| 204 | no hit |  |
| 205 | hypothetical protein FDH28_gp228 [Ralstonia phage RP12] | YP_009598886.1 |
| 206 | no hit |  |
| 207 | hypothetical protein FDH28_gp226 [Ralstonia phage RP12] | YP_009598888.1 |
| 208 | hypothetical protein FDH28_gp224 [Ralstonia phage RP12] | YP_009598890.1 |
| 209 | no hit |  |
| 210 | hypothetical protein FDH28_gp223 [Ralstonia phage RP12] | YP_009598891.1 |
| 211 | no hit |  |
| 212 | no hit |  |
| 213 | no hit |  |
| 214 | hypothetical protein FDH28_gp220 [Ralstonia phage RP12] | YP_009598894.1 |
| 215 | no hit |  |
| 216 | no hit |  |
| 217 | no hit |  |
| 218 | no hit |  |
| 219 | hypothetical protein FDH28_gp216 [Ralstonia phage RP12] | YP_009598898.1 |
| 220 | no hit |  |
| 221 | no hit |  |
| 222 | hypothetical protein FDH28_gp214 [Ralstonia phage RP12] | YP_009598900.1 |
| 223 | putative RNA ligase [Ralstonia phage RP12] | YP_009598902.1 |
| 224 | no hit |  |
| 225 | no hit |  |
| 226 | hypothetical protein FDH28_gp211 [Ralstonia phage RP12] | YP_009598903.1 |
| 227 | hypothetical protein FDH28_gp211 [Ralstonia phage RP12] | YP_009598903.1 |
| 228 | no hit |  |
| 229 | putative nicotinate phosphoribosyltransferase [Ralstonia phage RP12] | YP_009598904.1 |
| 230 | putative ribose-phosphate pyrophosphokinase [Ralstonia phage RP12] | YP_009598905.1 |
| 231 | no hit |  |
| 232 | no hit |  |
| 233 | hypothetical protein FDH28_gp206 [Ralstonia phage RP12] | YP_009598908.1 |
| 234 | no hit |  |
| 235 | no hit |  |
| 236 | no hit |  |
| 237 | no hit |  |
| 238 | hypothetical protein FDH28_gp201 [Ralstonia phage RP12] | YP_009598913.1 |
| 239 | no hit |  |
| 240 | no hit |  |
| 241 | no hit |  |
| 242 | no hit |  |
| 243 | hypothetical protein FDH28_gp197 [Ralstonia phage RP12] | YP_009598917.1 |
| 244 | hypothetical protein FDH28_gp196 [Ralstonia phage RP12] | YP_009598918.1 |
| 245 | no hit |  |
| 246 | no hit |  |
| 247 | no hit |  |
| 248 | hypothetical protein FDH28_gp192 [Ralstonia phage RP12] | YP_009598922.1 |
| 249 | no hit |  |
| 250 | putative virion structural protein [Ralstonia phage RP12] | YP_009598924.1 |
| 251 | hypothetical protein FDH28_gp189 [Ralstonia phage RP12] | YP_009598925.1 |
| 252 | putative virion structural protein [Ralstonia phage RP12] | YP_009598926.1 |
| 253 | hypothetical protein FDH28_gp187 [Ralstonia phage RP12] | YP_009598927.1 |
| 254 | hypothetical protein FDH28_gp186 [Ralstonia phage RP12] | YP_009598928.1 |
| 255 | hypothetical protein FDH28_gp185 [Ralstonia phage RP12] | YP_009598929.1 |
| 256 | hypothetical protein FDH28_gp184 [Ralstonia phage RP12] | YP_009598930.1 |
| 257 | hypothetical protein FDH28_gp182 [Ralstonia phage RP12] | YP_009598932.1 |
| 258 | hypothetical protein FDH28_gp181 [Ralstonia phage RP12] | YP_009598933.1 |
| 259 | hypothetical protein FDH28_gp180 [Ralstonia phage RP12] | YP_009598934.1 |
| 260 | putative virion structural protein [Ralstonia phage RP12] | YP_009598935.1 |
| 261 | putative virion structural protein [Ralstonia phage RP12] | YP_009598936.1 |
| 262 | putative virion structural protein [Ralstonia phage RP12] | YP_009598937.1 |
| 263 | putative virion structural protein [Ralstonia phage RP12] | YP_009598938.1 |
| 264 | putative virion structural protein [Ralstonia phage RP12] | YP_009598939.1 |
| 265 | no hit |  |
| 266 | hypothetical protein FDH28_gp174 [Ralstonia phage RP12] | YP_009598940.1 |
| 267 | hypothetical protein FDH28_gp173 [Ralstonia phage RP12] | YP_009598941.1 |
| 268 | hypothetical protein FDH28_gp172 [Ralstonia phage RP12] | YP_009598942.1 |
| 269 | putative virion structural protein [Ralstonia phage RP12] | YP_009598943.1 |
| 270 | putative T4-like DNA polymerase [Ralstonia phage RP12] | YP_009598944.1 |
| 271 | no hit |  |
| 272 | no hit |  |
| 273 | no hit |  |
| 274 | no hit |  |
| 275 | hypothetical protein FDH28_gp167 [Ralstonia phage RP12] | YP_009598947.1 |
| 276 | hypothetical protein FDH28_gp166 [Ralstonia phage RP12] | YP_009598948.1 |
| 277 | hypothetical protein FDH28_gp165 [Ralstonia phage RP12] | YP_009598949.1 |
| 278 | hypothetical protein FDH28_gp164 [Ralstonia phage RP12] | YP_009598950.1 |
| 279 | putative RNA polymerase beta prime subunit [Ralstonia phage RP12] | YP_009598951.1 |
| 280 | hypothetical protein FDH28_gp162 [Ralstonia phage RP12] | YP_009598952.1 |
| 281 | hypothetical protein FDH28_gp161 [Ralstonia phage RP12] | YP_009598953.1 |
| 282 | no hit |  |
| 283 | no hit |  |
| 284 | hypothetical protein FDH28_gp160 [Ralstonia phage RP12] | YP_009598954.1 |
| 285 | hypothetical protein FDH28_gp159 [Ralstonia phage RP12] | YP_009598955.1 |
| 286 | hypothetical protein FDH28_gp157 [Ralstonia phage RP12] | YP_009598957.1 |
| 287 | hypothetical protein FDH28_gp156 [Ralstonia phage RP12] | YP_009598958.1 |
| 288 | hypothetical protein FDH28_gp155 [Ralstonia phage RP12] | YP_009598959.1 |
| 289 | putative RAD2/SF2 helicase [Ralstonia phage RP12] | YP_009598960.1 |
| 290 | hypothetical protein FDH28_gp153 [Ralstonia phage RP12] | YP_009598961.1 |
| 291 | no hit |  |
| 292 | putative Putative RNA polymerase beta prime subunit [Ralstonia phage RP12] | YP_009598967.1 |
| 293 | putative DNA-directed RNA polymerase subunit beta [Ralstonia phage RP12] | YP_009598968.1 |
| 294 | hypothetical protein FDH28_gp145 [Ralstonia phage RP12] | YP_009598969.1 |
| 295 | hypothetical protein FDH28_gp144 [Ralstonia phage RP12] | YP_009598970.1 |
| 296 | hypothetical protein FDH28_gp143 [Ralstonia phage RP12] | YP_009598971.1 |
| 297 | hypothetical protein FDH28_gp142 [Ralstonia phage RP12] | YP_009598972.1 |
| 298 | hypothetical protein FDH28_gp141 [Ralstonia phage RP12] | YP_009598973.1 |
| 299 | hypothetical protein FDH28_gp140 [Ralstonia phage RP12] | YP_009598974.1 |
| 300 | putative Nuclease SbcCD, D subunit [Ralstonia phage RP12] | YP_009598975.1 |
| 301 | hypothetical protein FDH28_gp138 [Ralstonia phage RP12] | YP_009598976.1 |
| 302 | hypothetical protein FDH28_gp137 [Ralstonia phage RP12] | YP_009598977.1 |
| 303 | hypothetical protein FDH28_gp136 [Ralstonia phage RP12] | YP_009598978.1 |
| 304 | no hit |  |
| 305 | hypothetical protein FDH28_gp135 [Ralstonia phage RP12] | YP_009598979.1 |
| 306 | putative DNA-directed RNA polymerase subunit beta [Ralstonia phage RP12] | YP_009598980.1 |
| 307 | no hit |  |
| 308 | hypothetical protein FDH28_gp133 [Ralstonia phage RP12] | YP_009598981.1 |
| 309 | hypothetical protein FDH28_gp132 [Ralstonia phage RP12] | YP_009598982.1 |
| ^1^ e-value ≤ 1e−5 | |  |

| **Table S2.** Gene products encoded on genomic DNA of FLC4-3B and their closest hit searched with blastp in NCBI nr limited in *Caudoviricetes*. | | |
| --- | --- | --- |
| Gene products | The closest hits^1^ |  |
|  | Protein names [Virus strain] | Accession numbers |
| 1 | putative T4-like DNA polymerase [Ralstonia phage RP12] | YP_009598696.1 |
| 2 | no hit |  |
| 3 | hypothetical protein FDH28_gp004 [Ralstonia phage RP12] | YP_009598697.1 |
| 4 | hypothetical protein FDH28_gp005 [Ralstonia phage RP12] | YP_009598698.1 |
| 5 | hypothetical protein FDH28_gp006 [Ralstonia phage RP12] | YP_009598699.1 |
| 6 | hypothetical protein FDH28_gp007 [Ralstonia phage RP12] | YP_009598700.1 |
| 7 | hypothetical protein FDH28_gp008 [Ralstonia phage RP12] | YP_009598701.1 |
| 8 | hypothetical protein FDH28_gp009 [Ralstonia phage RP12] | YP_009598702.1 |
| 9 | hypothetical protein FDH28_gp010 [Ralstonia phage RP12] | YP_009598703.1 |
| 10 | no hit |  |
| 11 | hypothetical protein FDH28_gp011 [Ralstonia phage RP12] | YP_009598704.1 |
| 12 | hypothetical protein FDH28_gp012 [Ralstonia phage RP12] | YP_009598705.1 |
| 13 | hypothetical protein FDH28_gp013 [Ralstonia phage RP12] | YP_009598706.1 |
| 14 | hypothetical protein FDH28_gp014 [Ralstonia phage RP12] | YP_009598707.1 |
| 15 | no hit |  |
| 16 | hypothetical protein FDH28_gp016 [Ralstonia phage RP12] | YP_009598709.1 |
| 17 | no hit |  |
| 18 | hypothetical protein FDH28_gp017 [Ralstonia phage RP12] | YP_009598710.1 |
| 19 | hypothetical protein FDH28_gp018 [Ralstonia phage RP12] | YP_009598711.1 |
| 20 | hypothetical protein FDH28_gp019 [Ralstonia phage RP12] | YP_009598712.1 |
| 21 | hypothetical protein FDH28_gp020 [Ralstonia phage RP12] | YP_009598713.1 |
| 22 | hypothetical protein FDH28_gp021 [Ralstonia phage RP12] | YP_009598714.1 |
| 23 | hypothetical protein FDH28_gp022 [Ralstonia phage RP12] | YP_009598715.1 |
| 24 | hypothetical protein FDH28_gp023 [Ralstonia phage RP12] | YP_009598716.1 |
| 25 | putative terminase, large subunit [Ralstonia phage RP12] | YP_009598717.1 |
| 26 | putative virion structural protein [Ralstonia phage RP12] | YP_009598718.1 |
| 27 | putative virion structural protein [Ralstonia phage RP12] | YP_009598719.1 |
| 28 | hypothetical protein FDH28_gp027 [Ralstonia phage RP12] | YP_009598720.1 |
| 29 | putative tail shealth protein [Ralstonia phage RP12] | YP_009598722.1 |
| 30 | putative major virion structural protein [Ralstonia phage RP12] | YP_009598723.1 |
| 31 | no hit |  |
| 32 | no hit |  |
| 33 | hypothetical protein FDH28_gp031 [Ralstonia phage RP12] | YP_009598724.1 |
| 34 | no hit |  |
| 35 | hypothetical protein FDH28_gp032 [Ralstonia phage RP12] | YP_009598725.1 |
| 36 | hypothetical protein FDH28_gp034 [Ralstonia phage RP12] | YP_009598727.1 |
| 37 | hypothetical protein FDH28_gp036 [Ralstonia phage RP12] | YP_009598729.1 |
| 38 | putative virion structural protein [Ralstonia phage RP12] | YP_009598730.1 |
| 39 | hypothetical protein FDH28_gp038 [Ralstonia phage RP12] | YP_009598731.1 |
| 40 | hypothetical protein FDH28_gp039 [Ralstonia phage RP12] | YP_009598732.1 |
| 41 | hypothetical protein FDH28_gp040 [Ralstonia phage RP12] | YP_009598733.1 |
| 42 | putative RNA polymerase beta subunit [Ralstonia phage RP12] | YP_009598734.1 |
| 43 | putative RNA polymerase beta prime subunit [Ralstonia phage RP12] | YP_009598735.1 |
| 44 | hypothetical protein FDH28_gp043 [Ralstonia phage RP12] | YP_009598736.1 |
| 45 | hypothetical protein FDH28_gp044 [Ralstonia phage RP12] | YP_009598737.1 |
| 46 | hypothetical protein FDH28_gp046 [Ralstonia phage RP12] | YP_009598739.1 |
| 47 | hypothetical protein FDH28_gp047 [Ralstonia phage RP12] | YP_009598740.1 |
| 48 | hypothetical protein FDH28_gp048 [Ralstonia phage RP12] | YP_009598741.1 |
| 49 | hypothetical protein FDH28_gp049 [Ralstonia phage RP12] | YP_009598742.1 |
| 50 | no hit |  |
| 51 | hypothetical protein FDH28_gp051 [Ralstonia phage RP12] | YP_009598744.1 |
| 52 | hypothetical protein FDH28_gp052 [Ralstonia phage RP12] | YP_009598745.1 |
| 53 | hypothetical protein FDH28_gp053 [Ralstonia phage RP12] | YP_009598746.1 |
| 54 | putative virion structural protein [Ralstonia phage RP12] | YP_009598748.1 |
| 55 | putative GTP-binding protein [Ralstonia phage RP12] | YP_009598749.1 |
| 56 | no hit |  |
| 57 | no hit |  |
| 58 | hypothetical protein FDH28_gp058 [Ralstonia phage RP12] | YP_009598751.1 |
| 59 | putative UvsX protein [Ralstonia phage RP12] | YP_009598752.1 |
| 60 | hypothetical protein FDH28_gp060 [Ralstonia phage RP12] | YP_009598753.1 |
| 61 | hypothetical protein FDH28_gp061 [Ralstonia phage RP12] | YP_009598754.1 |
| 62 | putative ribonuclease H [Ralstonia phage RP12] | YP_009598755.1 |
| 63 | hypothetical protein FDH28_gp063 [Ralstonia phage RP12] | YP_009598756.1 |
| 64 | hypothetical protein FDH28_gp064 [Ralstonia phage RP12] | YP_009598757.1 |
| 65 | putative virion structural protein [Ralstonia phage RP12] | YP_009598758.1 |
| 66 | hypothetical protein FDH28_gp066 [Ralstonia phage RP12] | YP_009598759.1 |
| 67 | hypothetical protein FDH28_gp067 [Ralstonia phage RP12] | YP_009598760.1 |
| 68 | putative SbcC-ATPase [Ralstonia phage RP12] | YP_009598762.1 |
| 69 | no hit |  |
| 70 | no hit |  |
| 71 | no hit |  |
| 72 | hypothetical protein FDH28_gp070 [Ralstonia phage RP12] | YP_009598763.1 |
| 73 | putative virion structural protein [Ralstonia phage RP12] | YP_009598764.1 |
| 74 | hypothetical protein FDH28_gp072 [Ralstonia phage RP12] | YP_009598765.1 |
| 75 | hypothetical protein FDH28_gp073 [Ralstonia phage RP12] | YP_009598766.1 |
| 76 | no hit |  |
| 77 | hypothetical protein FDH28_gp082 [Ralstonia phage RP12] | YP_009598775.1 |
| 78 | no hit |  |
| 79 | hypothetical protein FDH28_gp084 [Ralstonia phage RP12] | YP_009598777.1 |
| 80 | hypothetical protein FDH28_gp085 [Ralstonia phage RP12] | YP_009598778.1 |
| 81 | putative crossover junction endodeoxyribonuclease RuvC [Ralstonia phage RP12] | YP_009598779.1 |
| 82 | putative virion structural protein [Ralstonia phage RP12] | YP_009598780.1 |
| 83 | putative virion structural protein [Ralstonia phage RP12] | YP_009598781.1 |
| 84 | putative virion structural protein [Ralstonia phage RP12] | YP_009598782.1 |
| 85 | no hit |  |
| 86 | hypothetical protein FDH28_gp091 [Ralstonia phage RP12] | YP_009598784.1 |
| 87 | putative RNA polymerase beta subunit [Ralstonia phage RP12] | YP_009598785.1 |
| 88 | hypothetical protein FDH28_gp093 [Ralstonia phage RP12] | YP_009598786.1 |
| 89 | putative major head protein [Ralstonia phage RP12] | YP_009598787.1 |
| 90 | hypothetical protein FDH28_gp095 [Ralstonia phage RP12] | YP_009598788.1 |
| 91 | putative DnaB helicase [Ralstonia phage RP12] | YP_009598789.1 |
| 92 | no hit |  |
| 93 | hypothetical protein FDH28_gp103 [Ralstonia phage RP12] | YP_009598796.1 |
| 94 | hypothetical protein FDG98_gp017 [Pseudomonas phage pf16] | YP_009595471.1 |
| 95 | no hit |  |
| 96 | hypothetical protein FDH28_gp100 [Ralstonia phage RP12] | YP_009598793.1 |
| 97 | no hit |  |
| 98 | no hit |  |
| 99 | hypothetical protein FDH28_gp105 [Ralstonia phage RP12] | YP_009598798.1 |
| 100 | hypothetical protein CF95_gp196 [Erwinia phage PhiEaH1] | YP_009010249.1 |
| 101 | hypothetical protein FDH28_gp106 [Ralstonia phage RP12] | YP_009598799.1 |
| 102 | no hit |  |
| 103 | no hit |  |
| 104 | no hit |  |
| 105 | no hit |  |
| 106 | no hit |  |
| 107 | no hit |  |
| 108 | hypothetical protein FDH28_gp111 [Ralstonia phage RP12] | YP_009598804.1 |
| 109 | no hit |  |
| 110 | no hit |  |
| 111 | hypothetical protein FDH28_gp114 [Ralstonia phage RP12] | YP_009598807.1 |
| 112 | no hit |  |
| 113 | hypothetical protein FDH28_gp115 [Ralstonia phage RP12] | YP_009598808.1 |
| 114 | hypothetical protein FDH28_gp116 [Ralstonia phage RP12] | YP_009598809.1 |
| 115 | hypothetical protein FDH28_gp117 [Ralstonia phage RP12] | YP_009598810.1 |
| 116 | hypothetical protein FDH28_gp118 [Ralstonia phage RP12] | YP_009598811.1 |
| 117 | hypothetical protein FDH28_gp119 [Ralstonia phage RP12] | YP_009598812.1 |
| 118 | no hit |  |
| 119 | hypothetical protein FDH28_gp121 [Ralstonia phage RP12] | YP_009598814.1 |
| 120 | hypothetical protein FDH28_gp120 [Ralstonia phage RP12] | YP_009598813.1 |
| 121 | no hit |  |
| 122 | hypothetical protein FDH28_gp124 [Ralstonia phage RP12] | YP_009598817.1 |
| 123 | hypothetical protein FDH28_gp125 [Ralstonia phage RP12] | YP_009598818.1 |
| 124 | no hit |  |
| 125 | no hit |  |
| 126 | no hit |  |
| 127 | hypothetical protein FDH28_gp130 [Ralstonia phage RP12] | YP_009598823.1 |
| 128 | no hit |  |
| 129 | no hit |  |
| 130 | no hit |  |
| 131 | hypothetical protein FDH28_gp289 [Ralstonia phage RP12] | YP_009598825.1 |
| 132 | hypothetical protein FDH28_gp288 [Ralstonia phage RP12] | YP_009598826.1 |
| 133 | putative deoxycytidine triphosphate deaminase [Ralstonia phage RP12] | YP_009598827.1 |
| 134 | no hit |  |
| 135 | no hit |  |
| 136 | hypothetical protein FDH28_gp285 [Ralstonia phage RP12] | YP_009598829.1 |
| 137 | hypothetical protein FDH28_gp284 [Ralstonia phage RP12] | YP_009598830.1 |
| 138 | hypothetical protein FDH28_gp277 [Ralstonia phage RP12] | YP_009598837.1 |
| 139 | no hit |  |
| 140 | no hit |  |
| 141 | no hit |  |
| 142 | no hit |  |
| 143 | no hit |  |
| 144 | no hit |  |
| 145 | no hit |  |
| 146 | hypothetical protein MZD04_gp268 [Pseudomonas phage Psa21] | YP_010347817.1 |
| 147 | no hit |  |
| 148 | putative concanavalin A-like protein [Ralstonia phage RSF1] | YP_009207894.1 |
| 149 | no hit |  |
| 150 | hypothetical protein AVU38_gp183 [Ralstonia phage RSL2] | YP_009213032.1 |
| 151 | hypothetical protein AVU38_gp184 [Ralstonia phage RSL2] | YP_009213033.1 |
| 152 | hypothetical protein AVU38_gp185 [Ralstonia phage RSL2] | YP_009213034.1 |
| 153 | no hit |  |
| 154 | hypothetical protein AVU11_gp168 [Ralstonia phage RSF1] | YP_009207972.1 |
| 155 | putative tail fiber protein [Ralstonia phage RP12] | YP_009598846.1 |
| 156 | hypothetical protein FDH28_gp267 [Ralstonia phage RP12] | YP_009598847.1 |
| 157 | hypothetical protein FDH28_gp271 [Ralstonia phage RP12] | YP_009598843.1 |
| 158 | no hit |  |
| 159 | hypothetical protein FDH28_gp269 [Ralstonia phage RP12] | YP_009598845.1 |
| 160 | putative tail fiber protein [Ralstonia phage RP12] | YP_009598846.1 |
| 161 | putative ribonucleotide reductase of class Ia (Aerobic), beta subunit [Ralstonia phage RP12] | YP_009598848.1 |
| 162 | putative ribonucleoside-diphosphate reductase [Ralstonia phage RP12] | YP_009598849.1 |
| 163 | no hit |  |
| 164 | hypothetical protein FDH28_gp263 [Ralstonia phage RP12] | YP_009598851.1 |
| 165 | hypothetical protein FDH28_gp262 [Ralstonia phage RP12] | YP_009598852.1 |
| 166 | hypothetical protein FDH28_gp261 [Ralstonia phage RP12] | YP_009598853.1 |
| 167 | no hit |  |
| 168 | no hit |  |
| 169 | putative DNA ligase [Ralstonia phage RP12] | YP_009598854.1 |
| 170 | no hit |  |
| 171 | hypothetical protein FDH28_gp259 [Ralstonia phage RP12] | YP_009598855.1 |
| 172 | no hit |  |
| 173 | no hit |  |
| 174 | hypothetical protein FDH28_gp256 [Ralstonia phage RP12] | YP_009598858.1 |
| 175 | putative transglycosylase [Ralstonia phage RP12] | YP_009598859.1 |
| 176 | putative phosphoesterase [Ralstonia phage RP12] | YP_009598861.1 |
| 177 | no hit |  |
| 178 | no hit |  |
| 179 | putative DEAD-like helicase [Ralstonia phage RP12] | YP_009598862.1 |
| 180 | no hit |  |
| 181 | no hit |  |
| 182 | no hit |  |
| 183 | no hit |  |
| 184 | hypothetical protein FDH28_gp245 [Ralstonia phage RP12] | YP_009598869.1 |
| 185 | no hit |  |
| 186 | no hit |  |
| 187 | hypothetical protein FDH28_gp242 [Ralstonia phage RP12] | YP_009598872.1 |
| 188 | no hit |  |
| 189 | no hit |  |
| 190 | hypothetical protein FDH28_gp241 [Ralstonia phage RP12] | YP_009598873.1 |
| 191 | hypothetical protein FDH28_gp240 [Ralstonia phage RP12] | YP_009598874.1 |
| 192 | no hit |  |
| 193 | no hit |  |
| 194 | no hit |  |
| 195 | no hit |  |
| 196 | hypothetical protein FDH28_gp237 [Ralstonia phage RP12] | YP_009598877.1 |
| 197 | hypothetical protein FDH28_gp236 [Ralstonia phage RP12] | YP_009598878.1 |
| 198 | no hit |  |
| 199 | hypothetical protein FDH28_gp234 [Ralstonia phage RP12] | YP_009598880.1 |
| 200 | hypothetical protein FDH28_gp233 [Ralstonia phage RP12] | YP_009598881.1 |
| 201 | hypothetical protein FDH28_gp232 [Ralstonia phage RP12] | YP_009598882.1 |
| 202 | no hit |  |
| 203 | hypothetical protein FDH28_gp231 [Ralstonia phage RP12] | YP_009598883.1 |
| 204 | no hit |  |
| 205 | hypothetical protein FDH28_gp228 [Ralstonia phage RP12] | YP_009598886.1 |
| 206 | no hit |  |
| 207 | hypothetical protein FDH28_gp226 [Ralstonia phage RP12] | YP_009598888.1 |
| 208 | hypothetical protein FDH28_gp224 [Ralstonia phage RP12] | YP_009598890.1 |
| 209 | no hit |  |
| 210 | hypothetical protein FDH28_gp223 [Ralstonia phage RP12] | YP_009598891.1 |
| 211 | no hit |  |
| 212 | no hit |  |
| 213 | no hit |  |
| 214 | hypothetical protein FDH28_gp220 [Ralstonia phage RP12] | YP_009598894.1 |
| 215 | no hit |  |
| 216 | no hit |  |
| 217 | no hit |  |
| 218 | no hit |  |
| 219 | hypothetical protein FDH28_gp216 [Ralstonia phage RP12] | YP_009598898.1 |
| 220 | no hit |  |
| 221 | no hit |  |
| 222 | hypothetical protein FDH28_gp214 [Ralstonia phage RP12] | YP_009598900.1 |
| 223 | putative RNA ligase [Ralstonia phage RP12] | YP_009598902.1 |
| 224 | no hit |  |
| 225 | no hit |  |
| 226 | hypothetical protein FDH28_gp211 [Ralstonia phage RP12] | YP_009598903.1 |
| 227 | hypothetical protein FDH28_gp211 [Ralstonia phage RP12] | YP_009598903.1 |
| 228 | no hit |  |
| 229 | putative nicotinate phosphoribosyltransferase [Ralstonia phage RP12] | YP_009598904.1 |
| 230 | putative ribose-phosphate pyrophosphokinase [Ralstonia phage RP12] | YP_009598905.1 |
| 231 | no hit |  |
| 232 | no hit |  |
| 233 | hypothetical protein FDH28_gp206 [Ralstonia phage RP12] | YP_009598908.1 |
| 234 | no hit |  |
| 235 | no hit |  |
| 236 | no hit |  |
| 237 | no hit |  |
| 238 | hypothetical protein FDH28_gp201 [Ralstonia phage RP12] | YP_009598913.1 |
| 239 | no hit |  |
| 240 | no hit |  |
| 241 | no hit |  |
| 242 | no hit |  |
| 243 | hypothetical protein FDH28_gp197 [Ralstonia phage RP12] | YP_009598917.1 |
| 244 | hypothetical protein FDH28_gp196 [Ralstonia phage RP12] | YP_009598918.1 |
| 245 | no hit |  |
| 246 | no hit |  |
| 247 | no hit |  |
| 248 | hypothetical protein FDH28_gp192 [Ralstonia phage RP12] | YP_009598922.1 |
| 249 | no hit |  |
| 250 | putative virion structural protein [Ralstonia phage RP12] | YP_009598924.1 |
| 251 | hypothetical protein FDH28_gp189 [Ralstonia phage RP12] | YP_009598925.1 |
| 252 | putative virion structural protein [Ralstonia phage RP12] | YP_009598926.1 |
| 253 | hypothetical protein FDH28_gp187 [Ralstonia phage RP12] | YP_009598927.1 |
| 254 | hypothetical protein FDH28_gp186 [Ralstonia phage RP12] | YP_009598928.1 |
| 255 | hypothetical protein FDH28_gp185 [Ralstonia phage RP12] | YP_009598929.1 |
| 256 | hypothetical protein FDH28_gp184 [Ralstonia phage RP12] | YP_009598930.1 |
| 257 | hypothetical protein FDH28_gp182 [Ralstonia phage RP12] | YP_009598932.1 |
| 258 | hypothetical protein FDH28_gp181 [Ralstonia phage RP12] | YP_009598933.1 |
| 259 | hypothetical protein FDH28_gp180 [Ralstonia phage RP12] | YP_009598934.1 |
| 260 | putative virion structural protein [Ralstonia phage RP12] | YP_009598935.1 |
| 261 | putative virion structural protein [Ralstonia phage RP12] | YP_009598936.1 |
| 262 | putative virion structural protein [Ralstonia phage RP12] | YP_009598937.1 |
| 263 | putative virion structural protein [Ralstonia phage RP12] | YP_009598938.1 |
| 264 | putative virion structural protein [Ralstonia phage RP12] | YP_009598939.1 |
| 265 | no hit |  |
| 266 | hypothetical protein FDH28_gp174 [Ralstonia phage RP12] | YP_009598940.1 |
| 267 | hypothetical protein FDH28_gp173 [Ralstonia phage RP12] | YP_009598941.1 |
| 268 | hypothetical protein FDH28_gp172 [Ralstonia phage RP12] | YP_009598942.1 |
| 269 | putative virion structural protein [Ralstonia phage RP12] | YP_009598943.1 |
| 270 | putative T4-like DNA polymerase [Ralstonia phage RP12] | YP_009598944.1 |
| 271 | no hit |  |
| 272 | no hit |  |
| 273 | no hit |  |
| 274 | no hit |  |
| 275 | hypothetical protein FDH28_gp167 [Ralstonia phage RP12] | YP_009598947.1 |
| 276 | hypothetical protein FDH28_gp166 [Ralstonia phage RP12] | YP_009598948.1 |
| 277 | hypothetical protein FDH28_gp165 [Ralstonia phage RP12] | YP_009598949.1 |
| 278 | hypothetical protein FDH28_gp164 [Ralstonia phage RP12] | YP_009598950.1 |
| 279 | putative RNA polymerase beta prime subunit [Ralstonia phage RP12] | YP_009598951.1 |
| 280 | hypothetical protein FDH28_gp162 [Ralstonia phage RP12] | YP_009598952.1 |
| 281 | hypothetical protein FDH28_gp161 [Ralstonia phage RP12] | YP_009598953.1 |
| 282 | putative concanavalin A-like protein [Ralstonia phage RSF1] | YP_009207894.1 |
| 283 | no hit |  |
| 284 | hypothetical protein FDH28_gp160 [Ralstonia phage RP12] | YP_009598954.1 |
| 285 | hypothetical protein FDH28_gp159 [Ralstonia phage RP12] | YP_009598955.1 |
| 286 | hypothetical protein FDH28_gp157 [Ralstonia phage RP12] | YP_009598957.1 |
| 287 | hypothetical protein FDH28_gp156 [Ralstonia phage RP12] | YP_009598958.1 |
| 288 | hypothetical protein FDH28_gp155 [Ralstonia phage RP12] | YP_009598959.1 |
| 289 | putative RAD2/SF2 helicase [Ralstonia phage RP12] | YP_009598960.1 |
| 290 | no hit |  |
| 291 | hypothetical protein FDH28_gp153 [Ralstonia phage RP12] | YP_009598961.1 |
| 292 | no hit |  |
| 293 | putative Putative RNA polymerase beta prime subunit [Ralstonia phage RP12] | YP_009598967.1 |
| 294 | putative DNA-directed RNA polymerase subunit beta [Ralstonia phage RP12] | YP_009598968.1 |
| 295 | hypothetical protein FDH28_gp145 [Ralstonia phage RP12] | YP_009598969.1 |
| 296 | hypothetical protein FDH28_gp144 [Ralstonia phage RP12] | YP_009598970.1 |
| 297 | hypothetical protein FDH28_gp143 [Ralstonia phage RP12] | YP_009598971.1 |
| 298 | hypothetical protein FDH28_gp142 [Ralstonia phage RP12] | YP_009598972.1 |
| 299 | hypothetical protein FDH28_gp141 [Ralstonia phage RP12] | YP_009598973.1 |
| 300 | hypothetical protein FDH28_gp140 [Ralstonia phage RP12] | YP_009598974.1 |
| 301 | putative Nuclease SbcCD, D subunit [Ralstonia phage RP12] | YP_009598975.1 |
| 302 | hypothetical protein FDH28_gp138 [Ralstonia phage RP12] | YP_009598976.1 |
| 303 | hypothetical protein FDH28_gp137 [Ralstonia phage RP12] | YP_009598977.1 |
| 304 | hypothetical protein FDH28_gp136 [Ralstonia phage RP12] | YP_009598978.1 |
| 305 | no hit |  |
| 306 | hypothetical protein FDH28_gp135 [Ralstonia phage RP12] | YP_009598979.1 |
| 307 | putative DNA-directed RNA polymerase subunit beta [Ralstonia phage RP12] | YP_009598980.1 |
| 308 | no hit |  |
| 309 | hypothetical protein FDH28_gp133 [Ralstonia phage RP12] | YP_009598981.1 |
| 310 | hypothetical protein FDH28_gp132 [Ralstonia phage RP12] | YP_009598982.1 |
| ^1^ e-value ≤ 1e−5 | |  |

[1] Horita M, Tsuchiya K (2000) Comparative analysis of Japanese and foreign strains of *Ralstonia solanacearum* based on 16S ribosomal RNA gene sequences. J Gen Plant Pathol 66:132-137
